# Therapeutical Relations of Intermittent Fever

**Published:** 1855-10

**Authors:** J. P. Hall

**Affiliations:** Glasgow, Ky.


### The Western Journal

OF

### MEDICINE AND SURGERY.

OCTOBER, 1855. NEW SERIES. VOL. IV.—No 4.

### ART. I.—THERAPEUTICAL RELATIONS OF INTERMITTENT FEVER.

BY J. P. HALL, M. D., OF GLASGOW, KY.

It will doubtless be thought by some a little remarkable, that in the present state of medical knowledge there should appear in a leading medical journal, a communication upon so rudimental a topic as the one I have selected. This subject cannot be said to have been neglected, yet so complete has been medical certainty in reference to the treatment of this disease, since the antiperiodic powers of cinchona were discovered, that few physicians have thought it needful to seek for a more efficient plan of arresting the paroxysms of intermitting fever, than was devised at a very early period after the alkaloid of Peruvian bark was brought into use.

It is evident that the curative power of quinine will display itself in this disease, no matter in what form or quantity, or in what stage of the disease administered, yet it is equally evident, to me, that the specific relations of the drug to the malady are developed in their highest degree, only when administered under special conditions. During a period of ten years, with tolerably extensive opportunities of experimenting in the management of this disease, I have pursued every possible modification of the quinine treatment, with the view of ascertaining as accurately as practicable, the conditions under which the highest remedial relations of the bark preparations are manifested. These observations have led to results highly satisfactory to myself, and in view of the probability that I may, in some degree, become instrumental in aiding, at least, younger members of the profession in escaping the perplexities, anxieties, and even disappointments which attended the first years of my own medical experience, I have thought I might confer a small service by committing some of my impressions to record.

Fourteen years from this time I made my professional debut at a small village situated on Barren river, in Warren county, and almost surrounded by an extensive body of fertile, alluvial bottom land. During the autumn of that year, intermitting fever was prevalent throughout Kentucky, and in the aforesaid bottom it was universal. After a brief, but spirited, combat with the disease in the persons of various of my new patrons, I was seized with a most relentless tertian myself. I was an intense sufferer during the ten or twelve days that followed, and well do I remember that my medical attendant watched over and administered to me most assiduously for well nigh this period before the arrestment of my "alternate day" paroxysms was accomplished. My case furnished a genuine specimen of tertian intermittent then as well as now. The cold stage was well marked, followed by prompt but rather violent reaction, and this stage terminating within from ten to twelve hours, in a gentle perspiration. Twenty-four hours, or more, of freedom from fever, with comparative comfort, intervening the paroxysms.

The treatment to which this case was subjected, consisted in active depletion—general blood-letting, emetics, mercurial purgatives, antimonials, &c., during the first three or four days, as preparatory, then the antiperiodic, quinine, in doses of one or two grains, repeated every second or third hour, during the apyrexial stage. This practice was not peculiar to the physician, whom I ought, perhaps, to remember with infinite gratitude, but it was the practice universally pursued by those around him, and the practice inculcated by those esteemed most trustworthy medical authorities, except in so far as these latter recommended a still farther complication of measures. This treatment is founded in views not compatible with the true therapeutical relations of the disease, and I have, long since, repudiated it in the main. The medical world has been not a little addicted to the error of confounding with the disease itself what are only its necessary concomitants—its sequences. Since the days of Sydenham and Ramazzini the idea has been prevalent that serious obstructions, engorgements, inflammations, &c., must be removed as a fixed condition to a safe resort to the measures appropriate for arresting the paroxysm—due antiphlogistic preparation of the system. Engorgements, hypertrophies, inflammations, effusions, indurations, &c., have been made to assume a false attitude, as respects exact pathological relationships and as respects a rational therapeutical diagnosis.

No matter what may be the nature of the agent to which the production of the disease is due; no matter in what manner or through what avenue the reception of this disease-producing element into the system is effected, it is a settled conviction of my mind that the primary morbid impression is upon the nervous system, and that this impression *de novo*, constitutes the disease; that the various functional and structural lesions, giving rise to their appropriate morbid expressions, are consecutive events, and due, not to the operation of the primary agent, but owe their development to a secondary impulse, and that these manifestations are *ordinarily* multiplied and aggravated in proportion as the paroxysm is repeated. The disease is in its essential form functional, and every organic complication which its progress involves constitutes a complication and of necessity something more than genuine intermittent fever.

Innervation and circulation—the nervous and vascular functions—are so mutually dependent for integrity of action, that the slightest disturbance of either necessarily deranges the other, so that the primary impression upon the former ultimately becomes so intense, as to produce *remora* of the blood and consequent suspension of calorification—chill. This is the earliest *characteristic* phenomenon exhibited in the disease, but one which creates a necessity for the development of subsequent phenomena. These depressing effects are antagonised by the re-active efforts of the organism—efforts that are salutary, only in so far as the restoration of heat and the circulation are concerned, while other functions remain suspended until farther, and still more salutary efforts are instituted for their restoration. This exaggerated state of matters subsides in a little while into a condition of things which displays nature's curative energies most strikingly in the various eleminative processes which are set up, tending to rid the economy of the primary morbific agent. Such efforts, unaided by art, are seldom completely adequate to the end, they prove sufficient only to effect such a diminution of the power of the vitiating agent, as to require the lapse of a given period before its impaired strength is again equal to the production of innervation so intensely perverted as to give rise to a new set of similar phenomena. Thus we have terminated the third or last stage of an intermittent paroxysm.

It is a question fairly admitting of discussion as to whether the duration of the period, constituting the interval, whether the anticipating or retarding tendency of the fits, be determined by the intrinsic virulence of the impressing agent, the result of the mere state of receptivity of the system at the period of exposure, or native impressibility of temperament. This inquiry involves questions to the solution of which I, at present, feel no disposition to address myself.

In my estimation it is in the third stage of an intermittent paroxysm, or the stage of declining excitement, alone that the natural tendencies to restoration are plainly exhibited. And the first and most important indication we have to fulfil, for the arrestment of the disease, is to aid and encourage these curative efforts already manifested by the economy. The office of remedies is not strictly to cure, but rather to support nature in her efforts to accomplish this end, and that material aid should be afforded by our remedial agents, they must be so administered as to stimulate the normal energies of the constitution just at the juncture when nature is struggling to counteract the morbific impulse; unless such co-operation is promoted, medical art is, in a great degree, exercised to no purpose, while nature's unaided efforts are seldom adequate to the complete and permanent arrestment of this peculiar malady. The first abnormal movement which we note, of a characteristic kind, is the chill; this we have said is the direct result of some—not very well understood—morbid impression upon the nervous tissue. These are about the first links wrought in the chain of morbid actions ensuant, and if the severance of these be effected before the extending chain becomes riveted to other systems, the full consummation of therapeutical interference is attained. To accomplish this we have but to induce a new impression upon the nervous tissue, and as this is promptly done, will be limited and rendered less formidable, consecutive evils requiring subsequent correction. We cannot, just here, too earnestly urge the momentousness of exercising a thorough discrimination, as to what constitutes the disease *essentially*, and what are its ulterior consequences, that we may not bestow our therapeutics in vain endeavors to repair consequences, while the primary and absolute disease which gave rise to them is left to an undisturbed progress. The numerous lesions which have already been cited, while they are the common sequelæ of advanced intermittents, do no more constitute essential elements in the pathology of this fever than does herpes labialis, or the ptyalisms sometimes induced by the barbarous treatment employed for its cure.

Although the experience of several years has resulted in no material modification of the opinions embodied in this communication, yet, until the observations furnished by the present extraordinary season, have been subjected to a diligent clinical analysis have I felt sufficiently warranted in their correctness to present them to the profession. Within a brief space, embracing only a few weeks, I have dispensed medicine to several hundred subjects of this disease, many of them assuming most anomalous types, presenting themselves under every conceivable mask. Urticaria, ophthalmia, diarrhœa, mere stupor and persistent somnolency, have in instances constituted the more obvious pathological manifestations, the only identifying feature being periodicity, but which, upon careful analysis of their history and nature, together with the prompt and satisfactory results of the anti-periodic treatment, were certainly enough traceable to their family origin. Why it is that such freaks are displayed in the history of this disease I am under no pledge to explain; such cases are not frequent, but certain it is, they occasionally present themselves, and

I am almost persuaded that the obstinacy of treatment, now and then evinced by skin, bowel and eye diseases, result from the fact that treatment is instituted in the absence of a correct diagnosis. While from the assumed semiology, we have an apparent case of urticaria, ophthalmia, or diarrhœa, the real disease, at least in its therapeutical relations, is intermittent fever. This apparent incongruity in these cases, would of itself, constitute an interesting inquiry and I think, judiciously treated. a profitable one, but the more regularly developed type of the disease must occupy my attention for the present.

In regard to the leading principles which have governed my therapeutical procedure, in this disease. I have uniformly endeavored to thwart the recurrence of the paroxysm, and have rarely had to witness its repetition after the exhibition of a first prescription. Periodicity has been the leading diagnostic element, as well as a controlling characteristic in regard to treatment. The fixed purpose of acting in concert with the efforts of nature, in re-establishing the normal tone of the nervous system, and relieving the obstructed secretions, has obtained throughout. Complicating events have seldom been absent in cases not seen until the paroxysm had been several times repeated, such as functional disturbances, visceral engorgements, topical inflammations, &c., but as a primary consideration, these have been disregarded, except in so far as it was convenient to treat them *pari passu*, in arresting the paroxysm. My treatment has been less obnoxious to the charge of complication than routineism, though such practice is fully justified in view of the routine nature of the disease itself.

As before intimated, I regard the sweating stage or stage of declining excitement, as the only period of the disease in which the economy manifests a salutary antagonism. When this stage is fairly past, quiescense succeeds such efforts, until it again supervenes, and I usually embrace it as the "golden moment" for administering the following prescription. (We are not in every instance opportunely consulted, if too early or too late, it should be made a point to await its return.)

Hydrarg. Pillu, twelve grains; Sub. mu. Mercury, six grains; Ext. Gentian, ten grains; Sulph. Quinine, fifteen grains. Incorporate the mass and form into pills. Immediately upon the appearance of decided perspiration, or, if no sweating supervenes, so soon as the fever has abated to this point, administer two thirds of the pills, and the remaining third four hours afterwards. There may have been no rigor or chill, there may not have been cool sensations sufficient to arrest the patient's attention, this is of little moment; periodicity, as respects pyrexial symptoms, must constitute the criterion. This prescription accomplishes the double office, as nature's abettor, of arresting the paroxysm, and disgorging the viscera of their excretive accumulations, just at the moment when the efforts of the organism are most active in consummating the same object. In a very large proportion of recent cases this prescription will complete the treatment, but in cases of more advanced standing, the consequences of protracted congestions and violent reactions, often repeated, demand more special care. In the event of an already relaxed state of the bowels, this combination may require a small astringent addition, or, if perchance, cathartics have been employed already, it may be proper to exclude the mercurial entirely. A failure to thwart the ensuing stages, which I guarantee will rarely happen, will render it necessary to administer another ten grain dose of quinine, upon the advent of the third stage. The observance of these rules may not always be practicable, as in cases without a frank development, or what will still more embarrass absolute rules, those vicarious intermittents, if I may so denominate them, previously alluded to. The tendency of this disease to relapse is great, perhaps greater than any other, almost, in the whole nosological list; the most rigid propriety upon the part of the patient is requisite to avert it. If such an occurrence is apprehended I know of nothing more certainly calculated to avert it than the exhibition of a very gentle purgative, combined with ten grains of quinine, on, from the seventh to the tenth day of convalescence. If cases of this disease were at times promptly and judiciously treated, there need be but little concern bestowed upon secondary lesions, but unfortunately the safe rule is reversed, and we consequently encounter such sequelæ, aggravated and obstinate in the extreme. I this day witnessed, in the person of a previously robust negroman, a case of general dropsy consequent upon the protraction of a neglected intermittent. These resulting troubles present themselves in such variety and extent as to form a wide field of investigation for the pathologico-therapeutical inquirer, and I am not forgetful either, that it has already been diligently and profitably cultivated. My present purpose will be more consistently conformed to by urging, at the expense of reiteration, the leading propositions which bear upon the disease in its essential and original form, in exclusion of events that owe their origin to its progress merely, and of necessity constitute adventitious features of the original. To sum up, I would insist upon the following as paramount propositions:—

1. Essential simplicity of the disease prior to the existence of secondary lesions, arising in its progress and directly the result of its continuance.

2. Importance of discriminating between the disease itself and its consequences merely, believing that these are often confounded to the prejudice of rational treatment.

3. Inutility of treatment, as preparatory to the arrestment of the paroxysm, as based upon the notiou that inflammatory engorgements, &c., exert a material agency in the causation of the paroxysm, and that the removal of the former is a necessary pre-requisite to the safe arrestment of the latter.

4. Advantage and economy of responding to the efforts of the organism, at the most propitious juncture for the employment of remedial agents.

5. The third, or stage of declining febrile excitement, being the only one in which such efforts are conspicuously displayed.

It may be thought that in urging objections to some of the pathological doctrines hitherto promulgated upon this subject, and the recommendations of treatment deduced therefrom, I have assumed a bold position, but the fact that the teachings of modern pathologists simply consist in the endorsement of opinions handed down to them, by an earlier medical generation, affords some apology for such a position. Authors have mostly given an unnecessary and forbidding complexity to the subject. The disease is essentially a simple disease, and as a subject of practical discussion it is susceptible of being presented in an aspect correspondingly devoid of complication. Khowledge, of book and lecture-room acquisition, constitutes, at a given period in the history of every medical man, the basis of his practical resources, and if this knowledge is to serve as his (only) guide in the discharge of his responsible duties in practice, rather than disconcert him at the bedside, simplicity should form the constant rule of those who assume the task of imparting it.

I trust these suggestions, offered as the result of bed-side impressions, may, in some degree, supply the absence of individual experience, in behalf of the young practitioner, for whom they are exclusively intended. I am persuaded that their adoption will insure to him, an easy and satisfactory career, so far as relates to the management of the simple disease, intermittent fever.

